# Optimized clonal isolation and immortalization of Rett syndrome patient fibroblasts for in vitro modeling of *MECP2 *mutations

**DOI:** 10.1038/s41598-025-19619-x

**Published:** 2025-10-13

**Authors:** Victoria Sarne, Anna Huber, Alexander V. Beribisky, Markus Hengstschläger, Franco Laccone, Hannes Steinkellner

**Affiliations:** 1 Institute of Medical Genetics, Center for Pathobiochemistry and Genetics, Medical University of Vienna, 1090 Vienna, Austria; 2 Nutritional and Sport Sciences (PhaNuSpo), Vienna Doctoral School of Pharmaceutical, University of Vienna, 1090 Vienna, Austria

**Keywords:** Neurodevelopmental disorders, Genetic models

## Abstract

Rett syndrome (RTT) is a severe neurodevelopmental disorder primarily caused by mutations in the X-linked *MECP2* gene. Patient-derived fibroblasts serve as a practical system to study systemic aspects of RTT, however, their limited proliferative capacity due to cellular senescence poses a significant challenge. In this study, we establish a robust workflow to isolate and immortalize clonal fibroblast lines from female RTT patients heterozygous for two distinct *MECP2* mutations (c.705delG and 1155del32). By employing single-cell cloning prior to hTERT-mediated immortalization, we generated stable, proliferative fibroblast clones with verified clonality and severely skewed X-chromosome inactivation. Wildtype clones exclusively expressed full-length MeCP2 protein, whereas mutant clones exhibit truncated or absent MeCP2. Immortalized lines retained elevated *hTERT* expression and sustained proliferation even at late passages. Notably, mutant clones recapitulated key molecular features of RTT, including histone hyperacetylation, dysregulation of oxidative stress markers, and aberrant expression of key signaling genes. Our approach provides a scalable and renewable in vitro model that faithfully captures critical aspects of RTT pathology and offers a complementary platform to existing animal and iPSC based systems. Moreover, the approach is adaptable for studying other X-linked genetic disorders and supports applications in mechanistic research and preclinical drug screening.

**Supplementary Information:**

The online version contains supplementary material available at 10.1038/s41598-025-19619-x.

## Introduction

Rett Syndrome (RTT) is a rare, severe neurodevelopmental disorder, mainly affecting females with a prevalence of 1 in 10,000 live female births^1^. RTT is characterized by developmental regression, loss of speech and purposeful hand use, and impairment of cognitive and motor skills. The disease is primarily caused by pathogenic variants in the X-linked *MECP2* gene^2^. This gene encodes the methyl-CpG binding-protein 2 (MeCP2), which is known as a universal activator and repressor of transcription^3^. Two isoforms of MeCP2 are generated through alternative splicing of the four *MECP2* exons^4^. Skipping of exon 2 produces the MeCP2-e1 isoform, which is predominantly expressed in the central nervous system (CNS) and is considered the RTT-relevant variant. Inclusion of all four exons results in translation initiation at exon 2 and production of the MeCP2-e2 isoform, which is more abundantly expressed in peripheral tissues^5^. The two isoforms are nearly identical except for their distinct N-terminal sequences^6^. Over 800 variants in the *MECP2* gene are so called RTT-causing *MECP2* mutations^7^. Notably, the severity of the phenotype often correlates with the specific type of mutation^8^ whereas the pattern of X chromosome inactivation does not necessarily show such correlation^9^.

While symptomatic treatments such as trofinetide^10,11^, an N-terminal tripeptide of the Insulin-like growth factor 1 (IGF1) protein, have recently become available, curative therapies remain elusive. Trofinetide partially alleviates core symptoms such as breathing problems, hand behaviors and anxiety^12^ but does not address the underlying molecular pathology of RTT. Given the lack of curative options, there is a continued need for reliable, accessible disease models to investigate RTT pathogenesis and support therapeutic development.

Most animal studies on RTT have been conducted in mice, ranging from *Mecp2* null mice^13–15^ to mouse models carrying human point mutations in the *Mecp2* gene^16–18^. Likewise, many cellular models rely on patient-derived induced pluripotent stem cells (iPSCs)^19,20^ or brain organoids^21,22^. While iPSC-based models allow sophisticated neuronal differentiation, they are technically demanding, time-consuming, and cost-intensive, limiting their accessibility for high-throughput or longitudinal studies.

A relatively simple and scalable approach is the generation of clonal mutant and wildtype human dermal fibroblasts (HDFs) from female RTT patients via single-cell seeding^23,24^. Despite their non-neuronal identity, fibroblasts obtained from RTT patients are valuable for investigating systemic cellular processes such as histone hyperacetylation, oxidative stress, and mitochondrial dysfunction that are relevant to RTT pathology^25–28^. However, these models face a critical limitation due to progressive senescence during serial passaging^29^ which severely restricts their long-term use and experimental flexibility. To address this issue, stable immortalization by transfection with a human telomerase reverse transcriptase (hTERT) plasmid provides a feasible strategy to extend the lifespan of patient-derived fibroblasts without compromising key cellular features^30^.

Here, we present a robust and high-throughput compatible cell model using immortalized clonal fibroblasts that complements existing ﻿in vivo﻿ and iPSC-based approaches. We hypothesized that hTERT-mediated immortalization can overcome replicative senescence in clonal RTT fibroblasts while preserving disease-relevant molecular signatures. This study aimed to develop an immortalized clonal fibroblast model of RTT, validate its molecular fidelity to disease-relevant features, and demonstrate its utility as a platform for preclinical testing.

Immortalized fibroblasts from RTT patients exhibit hallmark pathological features such as hyperacetylation^31–34^ and oxidative stress^35–37^ thereby offering a robust in vitro model for studying disease mechanisms and preclinical drug testing. Moreover, the establishment protocol described here can be readily adapted for the study of other X-linked diseases, providing a versatile tool for broader research applications.

## Results

### Clonal isolation is enhanced by workflow optimization

**Fig. 1:**
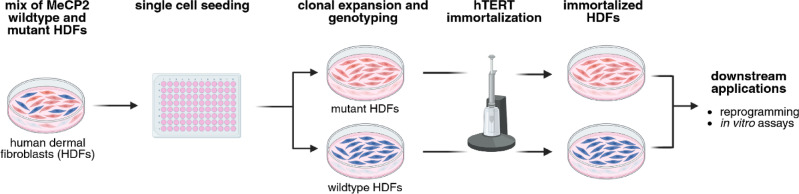
Clonal isolation and immortalization approach. Human dermal fibroblasts (HDFs) derived from RTT patients contain a mixture of cells expressing wildtype (blue) and mutant (red) MeCP2 protein. Primary mixed cultures were subjected to single-cell seeding, clonal expansion, and subsequent immortalization using the NEON electroporation system and an hTERT-expressing plasmid. Immortalized HDF clones were selected and further expanded, resulting in paired wildtype and mutant cell lines originating from the same patient.

Initial attempts to isolate clones by immortalizing mixed RTT fibroblast cultures prior to single-cell seeding were unsuccessful, yielding no proliferative wildtype or mutant clones. To overcome this limitation, we revised the workflow by performing single-cell seeding and clonal expansion before introducing hTERT-mediated immortalization. This adapted strategy enabled successful isolation and expansion of both wildtype and mutant clones from two independent RTT fibroblast lines (Fig. 1), carrying the c.705delG and the 1155del32 mutations.

Following clonal expansion, we confirmed the clonality and MeCP2 genotype of each cell line to ensure their suitability for downstream molecular and functional analysis.

### Clonal validation of MeCP2 expression and X-inactivation status

**Fig. 2:**
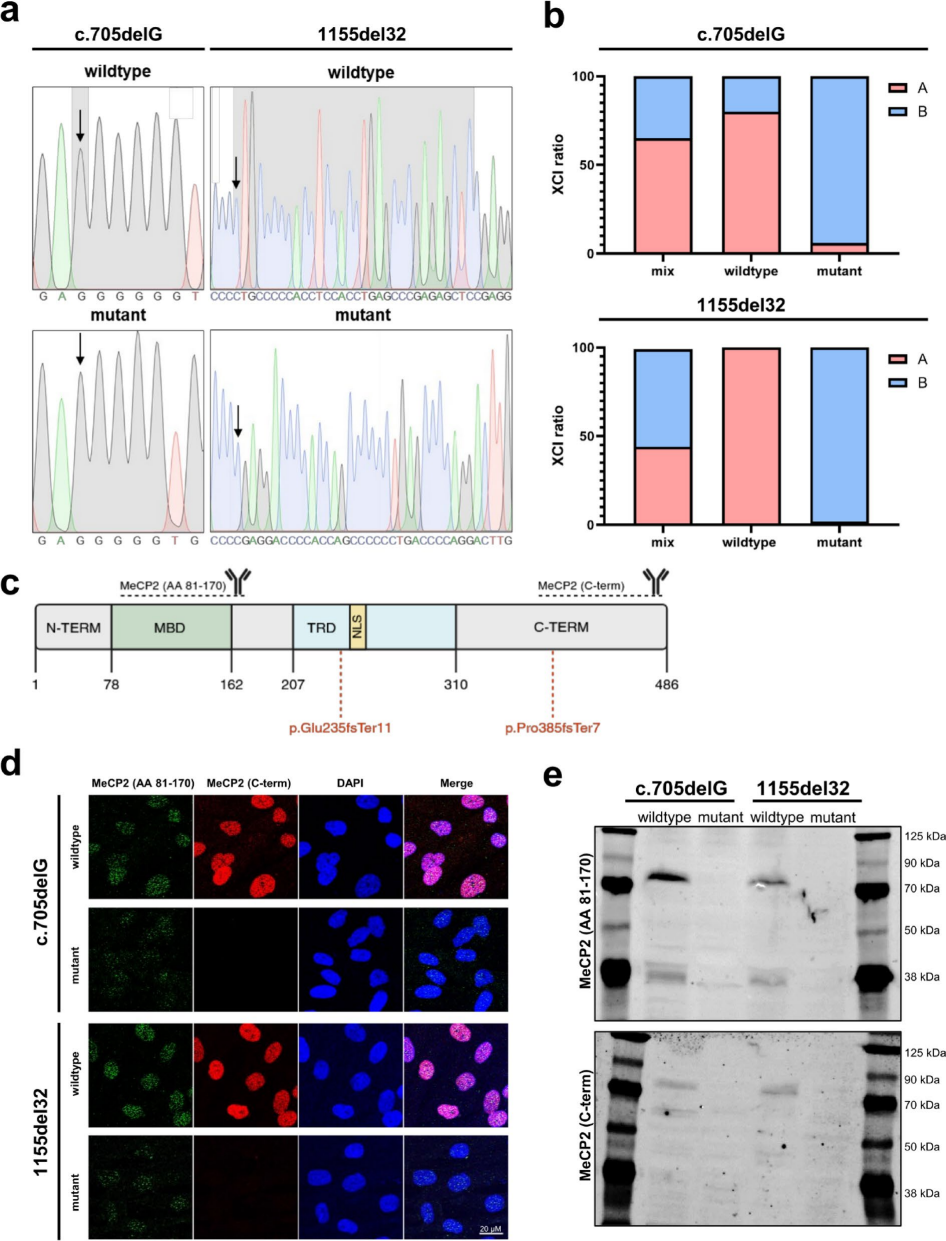
MeCP2 expression and X-chromosome inactivation (XCI) analysis of clones. The immortalized clones were analyzed for MeCP2 status via sequencing and HUMARA assay. (**a**) Sequencing of PCR products from amplified RNA shows that the clones derived from fibroblast lines harboring known *MECP2* mutations (c.705delG or 1155del32) express either wildtype or mutant MeCP2 transcripts. (**b**) XCI patterns were assessed using the HUMARA assay. Following HpaII digestion of active (non-methylated) X-chromosomes, PCR amplification and capillary gel electrophoresis were performed. The parental cell lines exhibit relatively balanced XCI, whereas the derived clones show skewed XCI, with only one active X-chromosome per clone. Chromosomes A and B are labelled arbitrarily. Representative chromatograms from the HUMARA assay are provided in Suppl. Figure 1. (**c**) Protein structure of MeCP2-e2. The pathogenic variant p.Glu235fsTer11 (c.705delG) is located at position 235, p.Pro385fsTer7 (1155del32) at position 385. MBD: methyl-CpG-binding domain, TRD: transcriptional repressor domain, NLS: nuclear localization signal (adapted from Huber et al. (2024)^34^. (**d**) In immunofluorescence imaging, both cell lines show MeCP2 expression in the wildtype clones, but not in the mutant clones using a C-terminal MeCP2 antibody. While some staining with an antibody targeting MeCP2 (AA 71–180) is detectable in the mutant clones, its intensity is considerably reduced. (**e**) Western blot analysis of MeCP2 shows full length protein only in the wildtype clones with both MeCP2 antibodies. Uncropped images of panel (**e**) are shown in Suppl. Figure 2.

To confirm the genetic and epigenetic integrity of the isolated clones, we evaluated MeCP2 expression at both the RNA and protein levels, as well as X-inactivation (XCI) status. Given that RTT patient-derived fibroblast clones are genetically identical except for their X-inactivation, we first assessed allele-specific MeCP2 expression using RT-PCR followed by Sanger sequencing. Wildtype clones expressed the wildtype MeCP2 transcript, while mutant clones exclusively expressed the mutant variant corresponding to their respective *MECP2* mutations (Fig. 2a). To further confirm clonality, XCI status was assessed using the HUMARA assay. While the original mixed fibroblast cultures exhibited relatively balanced XCI, both wildtype and mutant clonal lines showed highly skewed XCI, with expression from only one active X chromosome, consistent with monoclonal origin (Fig. 2b, Suppl. Figure 1).

We next assessed MeCP2 protein expression using immunofluorescence and western blotting with two MeCP2 antibodies: one antibody recognizing the epitope spanning amino acids (AA) 81–170 (upstream of the mutations), and another directed against the C-terminal region (downstream of the mutation sites). The mutations studied are located at amino acid positions 235 (c.705delG) and 385 (1155del32). Thus, the investigated mutations are located downstream of the epitope, where the MeCP2 (AA 81–170) antibody binds. In contrast, the C-terminal antibody binds downstream of the mutations and, as a result, no signal is observed (Fig. 2c).

Immunofluorescence of the MeCP2 antibody binding to amino acids 81–170 showed nuclear localization in both wildtype and mutant clones, indicating translation of the corresponding MeCP2 fragment. However, mutant clones exhibited markedly reduced staining intensity, consistent with truncated protein products. In contrast, the C-terminal antibody yielded nuclear signals only in wildtype clones, confirming the presence of full-length MeCP2 protein exclusively in these cells (Fig. 2d). Western blot analysis corroborated these findings, detecting full-length MeCP2 only in wildtype clones (Fig. 2e). Although western blotting is generally more sensitive compared to immunofluorescence, the absence of detectable truncated MeCP2 in mutant clones is likely due to degradation of the inherently unstable mutant protein during sample processing, including heat denaturation at 95 °C prior to SDS-PAGE. In contrast, immunofluorescence preserves in situ protein localization and stability, allowing detection of the truncated forms (p.Glu235fsTer11 and p.Pro385fsTer7). Taken together, these data validate the clonal identity of the fibroblast lines, confirm their X-inactivation-dependent MeCP2 expression profiles, and demonstrate their reliability for downstream functional analyses.

### Immortalized clones retain proliferative capacity and hTERT expression

**Fig. 3:**
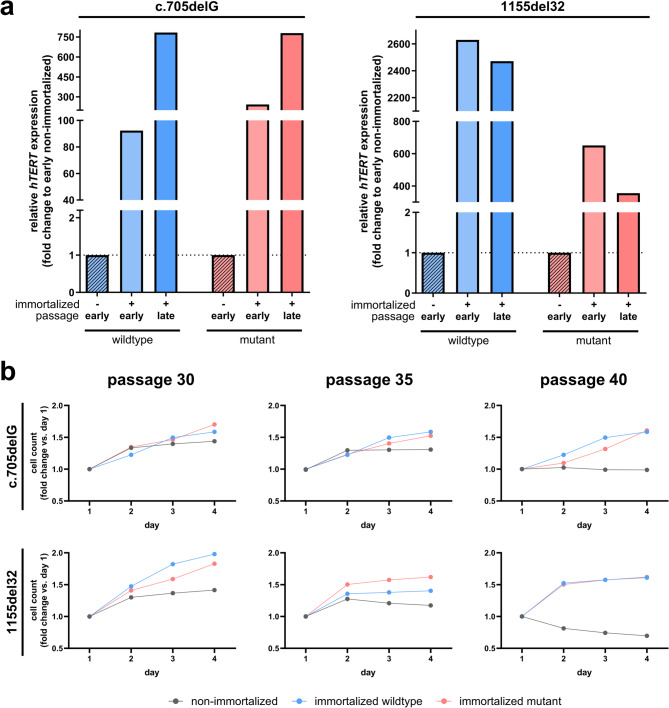
Immortalized RTT fibroblast clones retain proliferative capacity and high *hTERT* expression. (**a**) qPCR analysis of* hTERT* expression in control (non-immortalized) and immortalized fibroblast clones at early (passage ~ 20) and late (passage 30+) passages. Both wildtype and mutant immortalized clones from two independent RTT patient lines show a strong and sustained increase in *hTERT* expression compared to controls. Data are presented as fold change relative to early passage controls. (**b**) Proliferation analysis of immortalized and control fibroblast clones at passages 30, 35, and 40. Immortalized clones maintain robust proliferative capacity at late passages, whereas control cells cease proliferation by these passages. Representative growth curves are shown for each cell line and condition.

Next, we examined the proliferative capacity of the immortalized clones, as a key characteristic of their stability and potential for long-term use. Following hTERT-mediated immortalization, fibroblast cell lines were continuously passaged alongside non-immortalized controls. qPCR analysis of *hTERT* expression was assessed at early (approximately passage 20) and late (passage 30+) stages. Both immortalized cell lines exhibited a robust sustained upregulation of *hTERT* gene expression compared to early-passage non-immortalized controls (Fig. 3a).

To further evaluate the functional consequences of immortalization, we assessed the proliferation potential of fibroblast clones at passages 30, 35, and 40. Both immortalized cell lines retained their proliferation potential across all tested passage numbers, whereas control cells exhibited proliferative arrest by this stage. These findings confirm that hTERT immortalization effectively extends the replicative lifespan of RTT fibroblast clones while preserving their proliferative potential.

### Functional plasticity of immortalized clones demonstrated by neural progenitor transdifferentiation

To further validate the versatility of our immortalized fibroblast clones, we assessed their capacity for direct lineage reprogramming into induced neural progenitors (iNPs). Using a previously established protocol^34,38^ both wildtype and mutant clones from one RTT patient line were successfully transdifferentiated following transfection with neural reprogramming factors (SOX2, PAX6). Immunostaining confirmed expression of SOX2 and PAX6, and later-stage iNP clusters exhibited typical morphology along with robust expression of the neuronal progenitor markers Neurogenin-2 (NGN2) and Nestin (NES) (Suppl. Figure 3). These results demonstrate that our immortalized RTT fibroblast lines retain the plasticity necessary for neuronal lineage conversion, expanding their utility for downstream mechanistic studies and therapeutic screening in both non-neuronal and neuronal contexts.

### RTT-associated histone hyperacetylation and oxidative stress phenotypes are preserved in immortalized clones

To determine whether immortalized RTT fibroblast clones recapitulate hallmark molecular phenotypes, we evaluated histone acetylation levels, oxidative stress markers, and expression of key signaling genes.

**Fig. 4:**
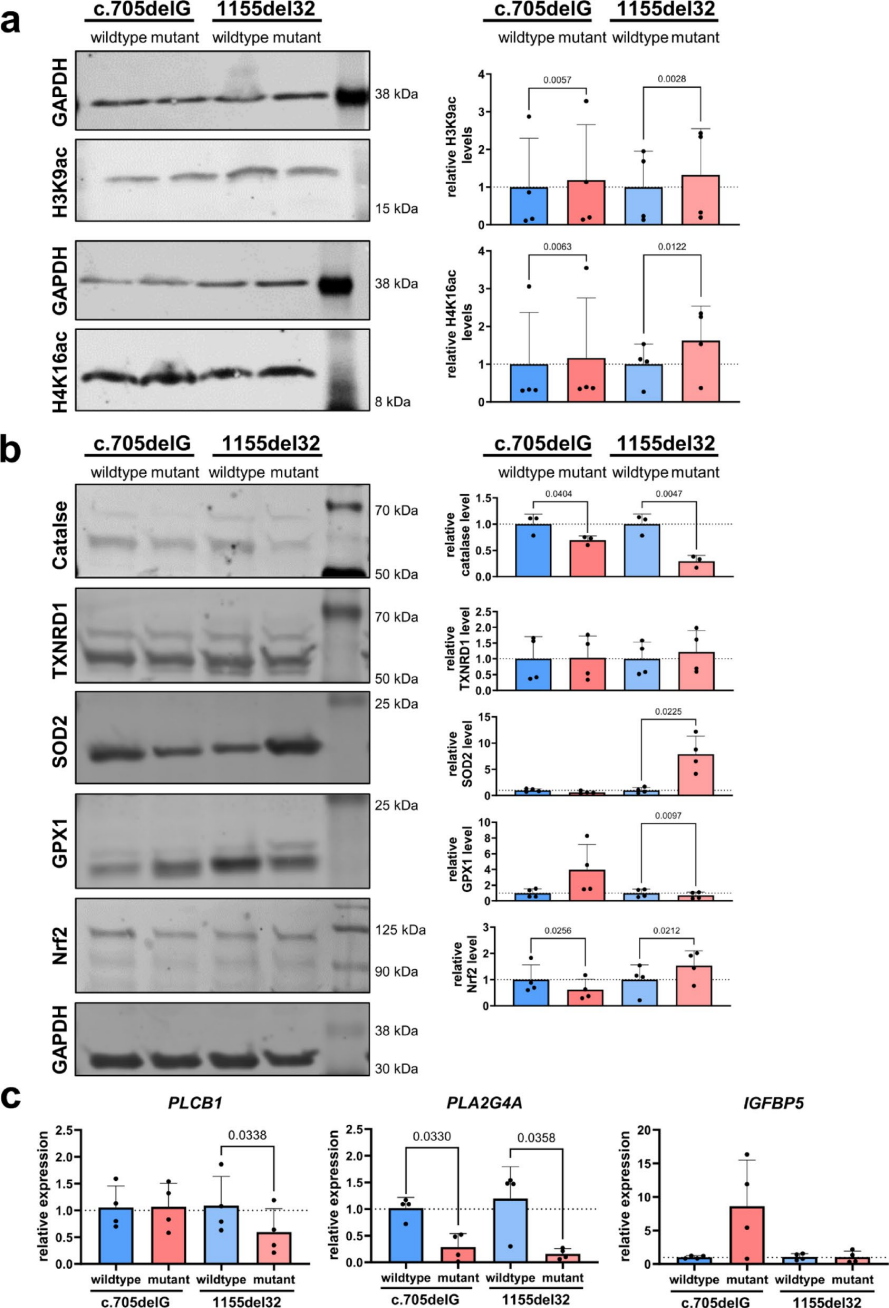
Immortalized RTT fibroblast clones recapitulate disease-associated hyperacetylation, oxidative stress, and gene expression changes. (**a**) Western blot analysis of H3K9ac and H4K16ac in isogenic wildtype and mutant clones derived from c.705delG and 1155del32 patient lines. Representative blots and quantification (normalized to GAPDH, shown as fold change relative to wildtype) are shown. Uncropped blots for panel (**a**) are provided in Suppl. Figure 4. Paired *t*-tests were performed comparing wildtype versus mutant clones: H3K9ac, c.705delG (*p* = 0.0057), 1155del32 (*p* = 0.0028); H4K16ac, c.705delG (*p* = 0.0063), 1155del32 (*p* = 0.0122) p values indicate significant differences. (**b**) Western blot analysis of catalase, TXNDR1, SOD2, GPX1, and Nrf2 in wildtype and mutant clones. Quantification is normalized to GAPDH and shown as fold change to wildtype. Uncropped images of panel (**b**) are shown in Suppl. Figure 5. Paired *t*-tests: Catalase, c.705delG (*p* = 0.0404), 1155del32 (*p* = 0.0047); TXNRD1, c.705delG (ns, *p* = 0.6561), 1155del32 (ns, *p* = 0.0673); SOD2, c.705delG (ns, *p* = 0.1959), 1155del32 (*p* = 0.0225); GPX1, c.705delG (ns, *p* = 0.1251), 1155del32 (*p* = 0.0097); and Nrf2, c.705delG (*p* = 0.0256), 1155del32 (*p* = 0.0212). (**c**) qPCR analysis of *PLCB1*, *PLA2G4A*, and *IGFBP5* expression in wildtype and mutant clones. Paired *t*-tests were performed for wildtype versus mutant comparisons: *PLCB1*, c.705delG (ns, *p* = 0.9372), 1155del32 (*p* = 0.0338); *PLA2G4A*, c.705delG (*p* = 0.0330), 1155del32 (*p* = 0.0358); and *IGFBP5*, c.705delG (ns, *p* = 0.1070), 1155del32 (*p* = 0.9649). Expression values are normalized to *PPIA* and shown as fold change to wildtype (ΔΔCt method). All data represent four independent biological replicates (separate lysates and harvests) per cell line and condition. Bars show mean ± SD. Ns = not significant.

For histone hyperacetylation (Fig. 4a), signal intensities for H3K9ac and H4K16ac were normalized to the housekeeping gene GAPDH and expressed relative to the mean of the isogenic wildtype controls. Statistical analysis using a paired *t*-test indicated significant increases in histone acetylation in RTT mutant clones compared to wildtype (c.705delG: H3K9ac: *p* = 0.0057; H4K16ac: *p* = 0.0063; 1155del32: H3K9ac: *p* = 0.0028, H4K16ac *p* = 0.0122). However, given the small sample size (*n* = 4 per group) and variability in the data, we additionally performed the non-parametric Mann-Whitney-U test. This analysis did not detect statistical significance. These findings highlight the exploratory nature of these results and are consistent with the modest changes in histone hyperacetylation reported in other RTT models^31–34,39^.

Analysis of oxidative stress markers revealed distinct genotype-specific profiles (Fig. 4b). In c.705delG mutant clones, protein levels of catalase and nuclear factor erythroid 2-related factor 2 (NRF2) were significantly reduced (*p* = 0.0404, *p* = 0.0256, respectively), while superoxide dismutase 2 (SOD2) and glutathione peroxidase (GPX1) showed non-significant changes. Thioredoxin reductase 1 (TXNDR1) remained unchanged. Conversely, in 1155del32 mutant clones, catalase was significantly decreased (*p* = 0.0047), whereas NRF2 and SOD2 was significantly upregulated (*p* = 0.0212, *p* = 0.0225, respectively). GPX1 expression was significantly downregulated (*p* = 0.0097) and TXNDR1 levels remained unchanged. All measurements were normalized to GAPDH and compared to isogenic wildtype controls.

qPCR analysis of signaling and stress-related genes further supported these findings (Fig. 4c). In c.705delG mutant clones, *PLA2G4A* gene expression levels were significantly downregulated (*p* = 0.0330), while *IGFBP5* expression was non-significantly elevated (*p* > 0.05). *PLCB1* did not show any changes in gene expression level. In contrast, 1155del32 mutant clones showed significant downregulation of *PLCB1* (*p* = 0.0338), *PLA2G4A* (*p* = 0.0358), while *IGFBP5* was unchanged compared to its wildtype counterpart.

## Discussion

In this study, we present a scalable and efficient approach for generating immortalized, isogenic clonal fibroblast lines from female RTT patients. This platform addresses a key limitation in primary fibroblast models - replicative senescence, which restricts their utility for long-term in vitro studies. By performing single-cell cloning prior to hTERT-mediated immortalization, we reliably established paired wildtype and mutant clones from two independent patient lines. The use of isogenic clones with defined XCI patterns minimizes genetic background variability, enabling direct comparison of *MECP2*-dependent phenotypes. Compared to mixed fibroblast cultures or iPSC-derived systems, our approach offers a genetically defined, expandable, and reproducible human model for mechanistic and therapeutic research in RTT^40^.

Clonal identity and genetic fidelity were confirmed by Sanger sequencing and XCI assays, which demonstrated monoclonal expression of either wildtype or mutant *MECP2* alleles. Consistent with these profiles, protein analysis revealed full-length MeCP2 expression and long term proliferative capacity in immortalized clones thereby confirming successful bypass of replicative senescence, extending the practical utility of these lines for continuous experimental use^41,42^.

Functional, immortalized mutant clones recapitulated key molecular features of RTT. Both MeCP2-mutant lines exhibited significant hyperacetylation of histone markers H3K9 and H4K16, consistent with epigenetic dysregulation observed in RTT tissues and in vitro models^31,43^. This highlights the relevance of fibroblasts in modeling systemic chromatin alterations in RTT, even outside the neuronal context^19^. Histone acetylation, particularly at H3K9 and H4K16, is a well-established marker of chromatin accessibility and transcriptional activity^44,45^. Increased acetylation at these residues has been linked to the loss of MeCP2-mediated transcriptional repression, as MeCP2 normally recruits histone deacetylases to maintain chromatin in a less accessible state^46,47^. Our findings are in line with previous reports of MeCP2-mediated chromatin remodeling and demonstrate that fibroblast models can effectively capture systemic epigenetic signatures of RTT, even in non-neuronal cell types^31,48^.

Analysis of oxidative stress markers revealed both shared and mutation-dependent changes. In c.705delG mutant clones, levels of catalase, NRF2 and SOD2 were consistently decreased, suggesting impaired redox homeostasis and antioxidant defense. In contrast, 1155del32 mutants showed divergent regulation of NRF2, GPX1 and SOD2, reflecting the complex oxidative imbalance and cellular heterogeneity characteristic across different *MECP2* mutations^35^ and underscore the importance of modeling multiple genotypes to fully capture the spectrum of RTT-related cellular dysfunction. The consistent downregulation of catalase and SOD2, key enzymes in hydrogen peroxide and superoxide detoxification^49,50^ is in line with previous reports linking oxidative stress to RTT pathology in patient tissues and mouse models^28,35^. However, potential secondary effects due to hTERT overexpression cannot be excluded. Previous studies have demonstrated a correlation between hTERT expression and enhanced antioxidant activity^51,52^. For instance, an upregulation of antioxidant enzymes, including catalase and superoxide dismutase, was observed in mesenchymal stem cells following hTERT overexpression^53^. Future investigations should include the non-immortalized HDFs lacking hTERT overexpression to more precisely delineate the direct impact of MeCP2 dysfunction on oxidative stress.

Gene expression profiling further supported the presence of RTT-associated molecular disruptions. Both mutant lines showed a significant downregulation of *PLA2G4A*, a gene involved in membrane lipid metabolism and signal transduction^54^. Its downregulation in both mutant lines may contribute to altered membrane dynamics and impaired cellular signaling, phenomena that have been implicated in RTT and other neurodevelopmental disorders^55,56^. Mutation-specific alterations in *PCLB1* and *IGFBP5* gene expression were also observed, consistent with their roles in signaling and stress response pathways in RTT and related disorders^23^. *PLCB1*, which encodes phospholipase C beta 1, a protein which plays a central role in phosphoinositide signaling and calcium homeostasis^57^. Reduced *PLCB1* expression in mutants may disrupt intracellular calcium signaling, a hallmark of RTT pathophysiology^19^. *IGFBP5*, or insulin-like growth factor binding protein 5, modulates IGF signaling and has been linked to cell survival and differentiation^58^. Interestingly, *IGFBP5* was increased in c.705delG mutants but remained relatively unchanged in 1155del32 mutants, suggesting mutation-specific effects on growth factor signaling. These data emphasize the potential of fibroblast models to unravel genotype-specific molecular consequences of *MECP2* dysfunction beyond the CNS.

Although fibroblasts lack neuronal identity, they offer a unique advantage as an accessible, renewable, and reprogrammable cell source. Recent work demonstrated that immortalized fibroblasts can be efficiently reprogrammed into induced neuronal progenitors (iNPs) and functional neurons^34^. This greatly expands the platform’s utility, enabling direct study of *MECP2* mutation effects in both non-neuronal and neuronal contexts from the same genetic background. This dual use is particularly relevant for RTT, which involves dysfunction in both neuronal and non-neuronal tissues. Our platform may therefore serve as a valuable foundation for integrated studies of RTT mechanisms and for screening of genotype-specific therapeutic candidates.

Despite its advantages, this model has several limitations. As non-neuronal cells, fibroblasts do not exhibit the neuron-specific gene expression programs central to RTT neuropathology. While reprogramming into neurons can partially address this gap, the fidelity of such induced phenotypes must be validated against primary CNS tissues or iPSC-derived neurons. Additionally, XCI skewing in clonal fibroblast lines may not fully replicate the mosaicism seen in vivo, and the two *MECP2* mutations studied here may not represent the full clinical heterogeneity of RTT. Further work incorporating additional patient lines and functional assays will be necessary to generalize these findings.

In summary, we present an immortalized clonal fibroblast platform that offers a reliable, accessible, and genetically defined tool for RTT research and therapeutic development. By combining genetic and epigenetic fidelity, extended proliferative capacity, and the ability to model both canonical and mutation-specific molecular phenotypes, this system complements existing animal and iPSC-based models. The workflow described here can also be adapted to other X-linked disorders. Future work could focus on expanding the range of *MECP2* mutations modeled, using multi-omics approaches for deeper mechanistic insights, and leveraging reprogramming strategies to explore neuronal phenotypes in vitro. Together, these advances may help to further clarify the pathogenesis of RTT and accelerate the development of targeted therapeutic therapies.

## Methods

### General cell culture

The following human dermal fibroblast (HDF) cell lines were obtained from the NIGMS Human Genetic Cell Repository at the Coriell Institute for Medical Research: 1155del32 (#GM11272) and c.705delG (#GM07982). Both lines were cultured in growth medium (DMEM supplemented with 15% fetal bovine serum (FBS) and 1% Penicillin/Streptomycin). Cell were passaged 1:3 every 3–4 days when the cultures reached about 90% confluence. Conditioned medium was collected after 72 h, sterile filtered and stored at −20 °C until needed.

### Single cell clone isolation

HDFs were seeded at a density of 0.9 cells/well in 96 well plates in a 1:1 mixture of fresh growth medium and conditioned medium. Medium was changed once per week. When a well reached 90% confluence, the cells were passaged into a 24 well plate. This process was repeated until sufficient cells for a T75 flask were reached. Once the flask reached confluence, immortalization was performed.

### Immortalization

Immortalization of clones was conducted using the NEON transfection system (Invitrogen). The plasmid pCl neo-hEST2 (#1781, Addgene) was used. For the electroporation, cells were trypsinized, collected, counted, and washed with DPBS. Pelleted cells were resuspended in NEON resuspension buffer at 1 × 10^6^ cells/100 µL. 15 ug of plasmid were used per transfection. A non-transfected control was seeded in parallel. Electroporation was carried out using 100 µL pipette tips of the Neon Transfection System (#MPK10096; Invitrogen) according to the manufacturer’s instructions. Electroporation conditions used were one pulse with 1,000 V for 60 ms. Electroporated cells were then seeded in polystyrene six-well plates containing 2 mL fibroblast proliferation medium and allowed to recover for 3 days. Post recovery, selection medium was applied (growth medium containing 2 mg/mL G418). The appropriate concentration was determined using a standard antibiotic kill curve. After two weeks, all cells in the non-transfected control had died, and the G418 concentration in the medium was reduced to 0.2 mg/mL.

### Mutation status analysis

To analyse the *MECP2* mutation status of clones, total RNA was isolated. Both wildtype and mutant expressing cells carry both variants on a genomic level, but one X-chromosome, on which MeCP2 is located, is inactivated. Hence, the isolation of RNA was necessary. RNA was transcribed into cDNA according to manufacturer’s instructions using SuperScript™ IV First-Strand Synthesis System (#18091050, Invitrogen). Subsequently, standard PCR was performed using the following primers: c.705delG: forward 5’-CGAGAGCAGAAACCACCTAA-3’ and reverse 5’-AGTCTTCTATCCGATCTGTGCAGGAG-3’; 1155del32: forward 5’-CGAGAGCAGAAACCACCTAA-3’ and reverse 5’-CAAAGACATTGTTTCATCCTC-3’. The resulting PCR product was sent for sequencing. Sequencing results revealed the appropriate mutation status.

### HUMARA assay

DNA of clones was isolated using the DNeasy Blood and tissue kit according to manufacturer’s instructions. HUMARA assay was conducted as described in Jones, 2014^59^.

### Immunofluorescence

HDFs were fixed with ice-cold 4% (vol/vol) formaldehyde in DPBS for 20 min at 4 °C and permeabilized in DPBS with 0.2% Triton X-100 twice for 5 min. After blocking for 30 min with 3% bovine serum albumin in DPBS, primary antibodies with 3% goat serum were applied overnight at 4 °C. Primary Antibodies used are α-MeCP2 C-terminal (#3456, Cell Signaling Technology; 1:100) and α-MeCP2 AA 81–170 (#SAB1404063, Sigma; 1:100). Before and after incubation with secondary antibodies (Goat anti-Mouse IgG Alexa Fluor 488 #11029, Goat anti-Rabbit IgG Alexa Fluor 568 #11011; Invitrogen, 1:250 in DPBS with 3% goat serum) for 1 h at room temperature, cells were washed with DPBS thrice for 5 min. Coverslips were mounted using ProLong Diamond Antifade Mountant with DAPI (#P36966; Invitrogen). All fluorescent images were acquired using a confocal microscope (#DMI6000; Leica Microsystems) and LASX 3.5 software. The following objectives were used: HC PL APO CS 20x/0.70 UV (#506513; Leica Microsystems) and HCX PL APO CS 40x/1.25 OIL PH3 UV (#506181; Leica Microsystems). Images were deconvolved with Huygens Essential version 23.04 (Scientific Volume Imaging; http://svi.nl).

### *hTERT* expression analysis

Telomerase activity was quantified using the Telomerase Activity Quantification qPCR Assay Kit (#8928, ScienCell). First, all cell pellets at the according passage numbers were collected and stored at −80 °C until all pellets of one cell line were collected. Subsequently, the assay was carried out according to manufacturer’s instructions. Non-transfected controls of clones could only be obtained for the early passage number samples, as cells stopped proliferating before reaching “late” passage numbers. *hTERT* expression was normalized to the non-immortalized fraction of each clone at the early passage number.

### Proliferation assay

The proliferation assay was carried out by seeding clones or original cell lines at certain passage numbers into 96 well plates at 5,000 cells/well. Cells were left to attach overnight and then stained with 5 µM Hoechst for 5 min. Cells were subsequently washed and fresh medium was applied. Cells of an area of 7.68 mm² were counted using the ImageXpress Pico imaging system and CellReporterXpress software immediately after staining and then in 24-hour intervals until 96 h. Cell counts were normalized to the first count and expressed as relative increase after. Averages of 6 wells per condition are shown.

### Hyperacetylation analysis

To investigate RTT hallmark hyperactylation in the immortalized clones, cells pellets were harvested at similar passage numbers and lysed in RIPA buffer to isolate total protein. Protein concentrations were determined using the Pierce BCA Protein Assay Kit (#23227, Thermo Scientific). Protein samples were separated on 12% SDS-Page gels and transferred to membranes using the iBlot System (Thermo Fisher Scientific). The membranes were blocked with Intercept (TBS) blocking buffer (#927-60001, Li-Cor) and incubated overnight at 4 °C with primary antibodies against H3K9ac (#H9286, Sigma), H4K16ac (#07-329, Sigma) and GAPDH (#MAB374, Merck). Membranes then were washed in TBST and the appropriate IRDye secondary antibodies (Li-Cor) and visualized using an ODYSSEY CLx imaging system. The band intensity was quantified using Image Studio Lite software with GAPDH as the loading control.

### Oxidative stress analysis

Oxidative stress analysis was performed via western blot as described in the previous section. The following antibodies were used against catalase (#66765-1-Ig, Proteintech), GPX1 (#29329-1-AP, Proteintech), SOD2 (#66474-1-Ig, Proteintech), TXNDR1 (#11117-1-AP, Proteintech) and Nrf2 (#ab137550, Abcam).

### Gene expression analysis

RNA was isolated and transcribed as described above. qPCR was conducted using TaqMan Probes against the target genes (*PBLC1* #Hs01001930_m1; *PLA2G4A* #Hs00996912_m1; *IGFPB5* #Hs00181213_m1; ThermoFisher) and iTaq Universal Probes Supermix (#172–5131, Bio-Rad) according to the manufacturer’s instructions. All qPCR values were normalized to *PPIA* and calculated as fold change relative to wildtype using the ΔΔCt method^60^. All experiments were performed on four independently acquired lysates per cell line and condition, and data are presented as mean ± SD. Statistical significance was determined by a paired *t*-test.

### Reprogramming

Reprograming to iNPs was conducted as previously described^34^. Briefly, HDFs were transfected with plasmids encoding *SOX2* and *PAX6* for reprogramming. Post-transfection, cells were maintained in neural reprogramming medium and regularly replated.

## Supplementary Information

Below is the link to the electronic supplementary material.

Supplementary Material 1

## Data Availability

The datasets generated and/or analyzed during the current study are partially included in the Supplementary Information files. Additional data supporting the findings of this study are available from the corresponding author upon reasonable request.
