# Supplementary material for: Optimized clonal isolation and immortalization of Rett syndrome patient fibroblasts for in vitro modeling of MECP2 mutations
Source: Sci Rep. 2025 Oct 13;15:35696. doi: 10.1038/s41598-025-19619-x (PMC12518718; doi:10.1038/s41598-025-19619-x)
Supplement: Supplementary file 1 — Supplementary Material 1 [file 41598_2025_19619_MOESM1_ESM.docx]

# Supplementary Material

Optimized Clonal Isolation and Immortalization of Rett Syndrome Patient Fibroblasts for *In Vitro* Modeling of *MECP2* Mutations

# Victoria Sarne1,2,+, Anna Huber1,+, Alexander V. Beribisky1, Markus Hengstschläger1, Franco Laccone1, and Hannes Steinkellner1,*

1Institute of Medical Genetics, Center for Pathobiochemistry and Genetics, Medical University of Vienna, Vienna, 1090, Austria

2Vienna Doctoral School of Pharmaceutical, Nutritional and Sport Sciences (PhaNuSpo), University of Vienna, Vienna, 1090, Vienna, Austria

*[hannes.steinkellner@meduniwien.ac.at](mailto:hannes.steinkellner@meduniwien.ac.at)

+these authors contributed equally to this work


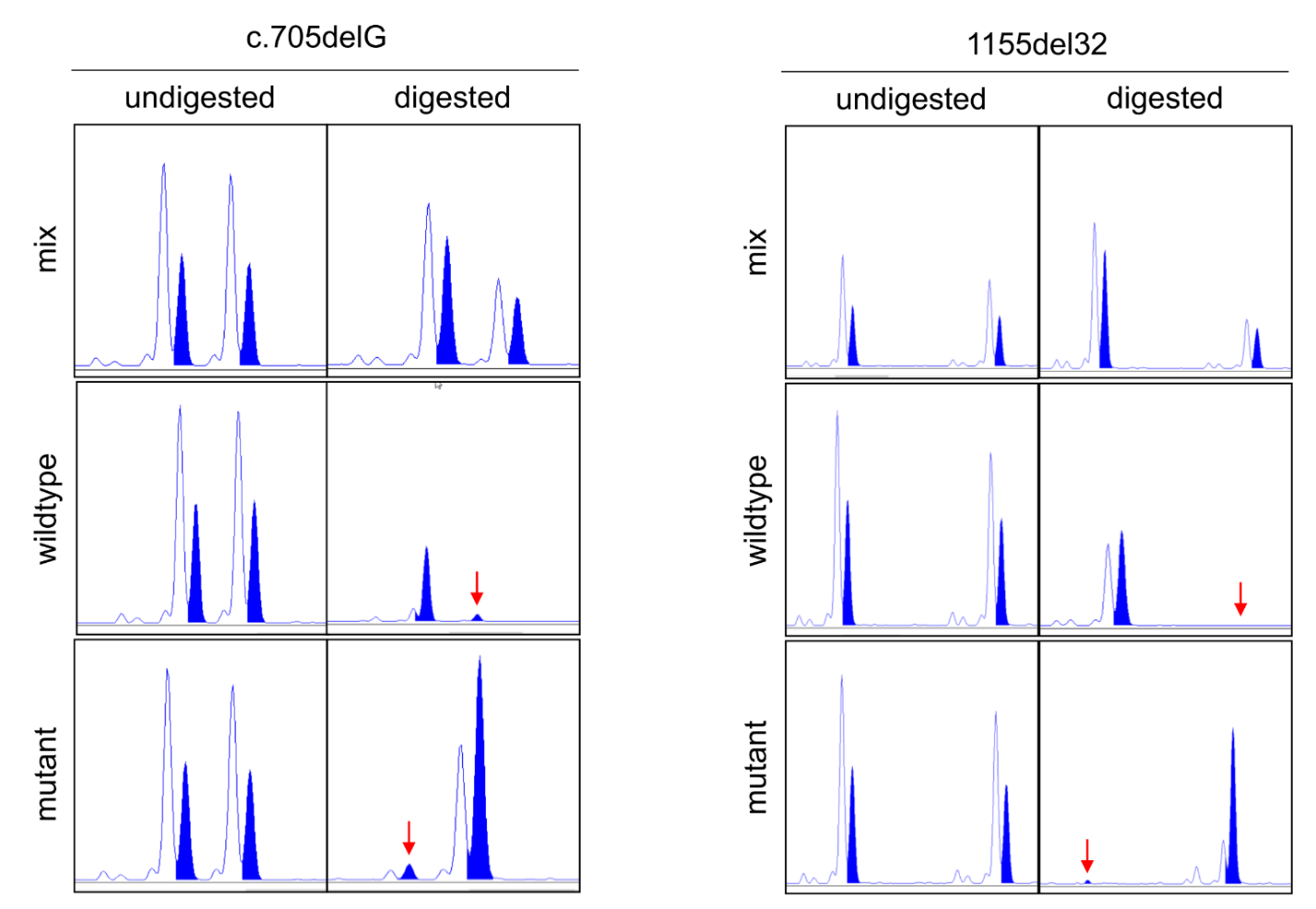


***S******upplementary Figure 1. Representative HUMARA assay chromatograms showing X-chromosome inactivation (XCI) patterns in clones.***

*XCI was analyzed using the HUMARA assay. Genomic DNA from parental cell lines and derived clones was either left undigested or digested with the methylation-sensitive restriction enzyme HpaII, which selectively cleaves unmethylated (active) X-chromosomes. This was followed by PCR amplification of the HUMARA locus and fragment analysis via capillary electrophoresis. Representative chromatograms are shown for two mutations (c.705delG and 1155del32), comparing undigested (left) and HpaII-digested (right) DNA from mixed, wildtype, and mutant cell populations. In undigested samples, both X-chromosome alleles are typically visible. Following digestion, only the methylated (inactive) X-chromosome is amplified. Parental (mixed) populations exhibit relatively balanced XCI, while the derived wildtype and mutant clones show skewed XCI, with expression from only one X-chromosome. Blue-filled peaks indicate the allele-specific PCR products used for quantification, corresponding to repeat lengths of 366 and 370 bp for c.705delG, and 370 and 384 bp for 1155del32. Red arrows denote the positions of signals markedly reduced upon digestion, indicating the presence of an active, unmethylated allele.*

#
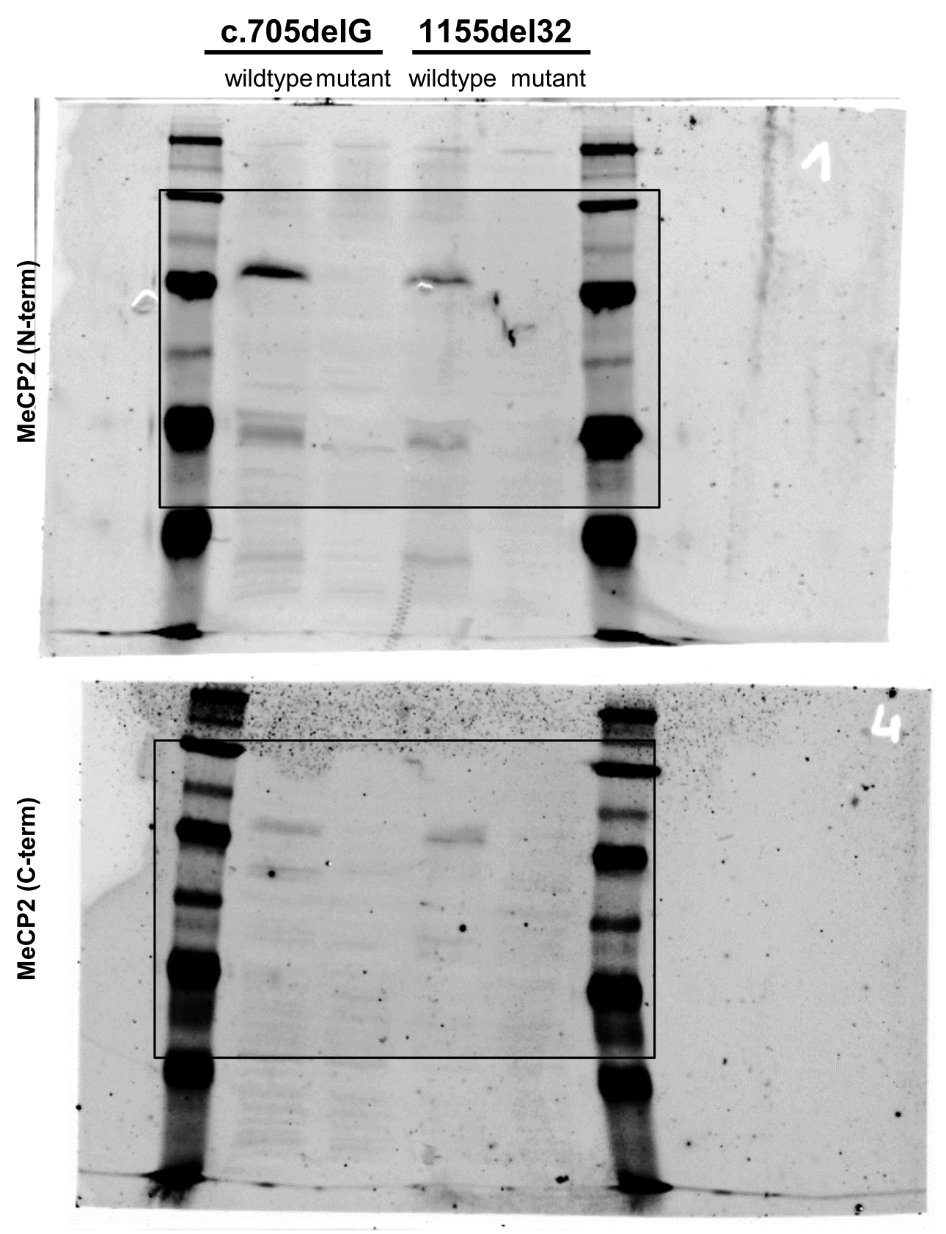


**Supplementary Figure 2. Uncropped images of the immunoblots shown Fig. 2e.**

#
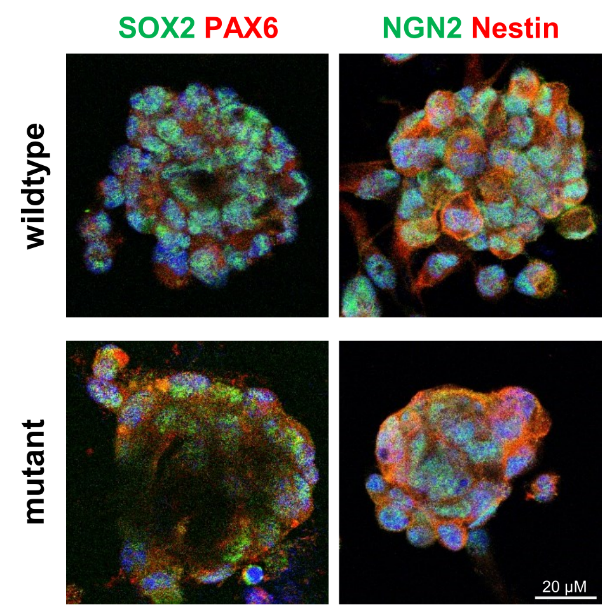


**Supplementary Figure 3. Immunofluorescence analysis of induced neural progenitor (iNP) clusters derived from immortalized wildtype and mutant fibroblast clones of c.705delG cells.** Cells were transdifferentiated using neural reprogramming factors as described (Huber et al., 2024). Representative images show nuclear expression of SOX2 and PAX6 in early reprogrammed cells, and NGN2 and NESTIN expression in mature iNP clusters with typical morphology. Cells were counterstained with DAPI (blue).


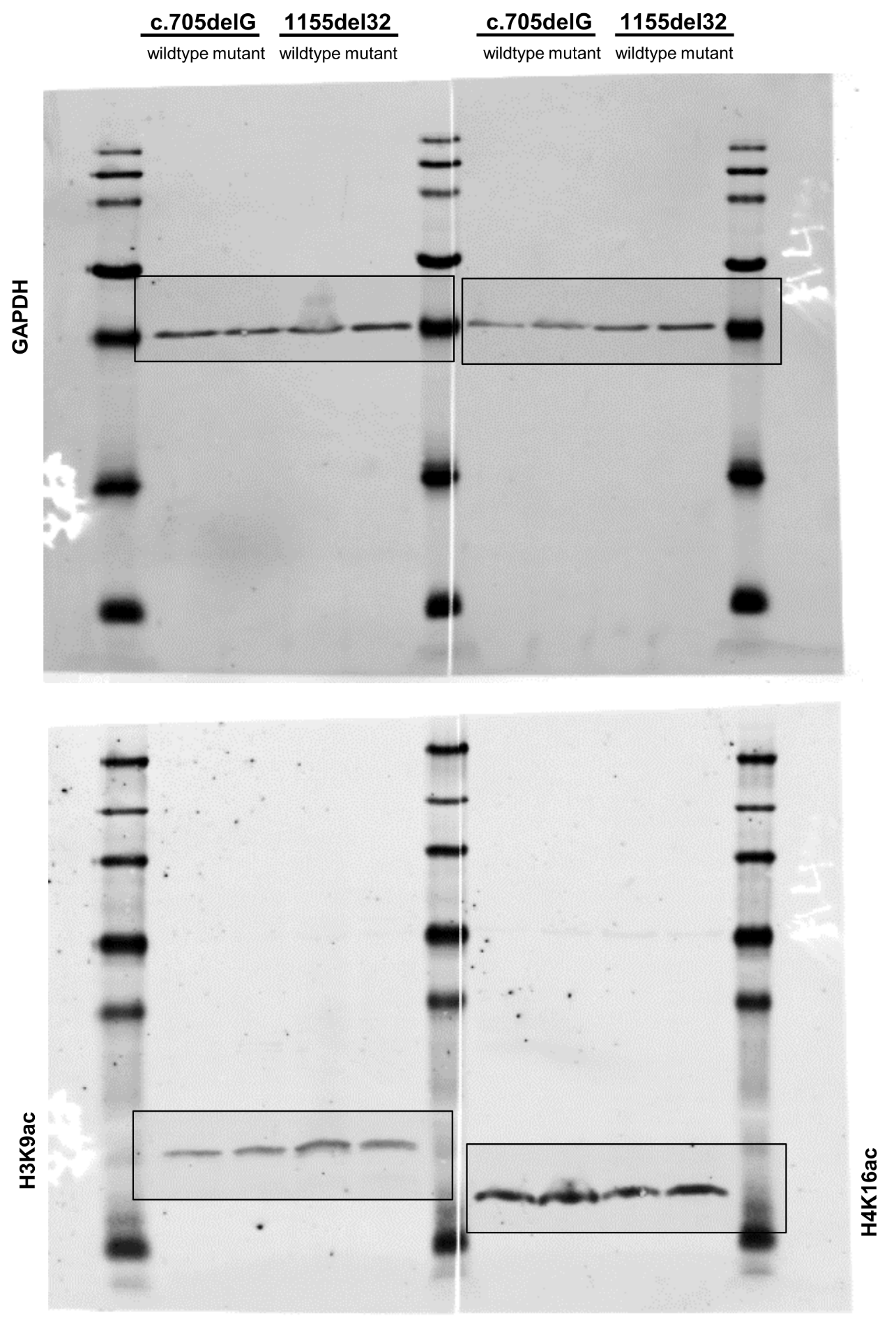


**Supplementary Figure 4. Uncropped images of the immunoblots shown Fig. 4a.**


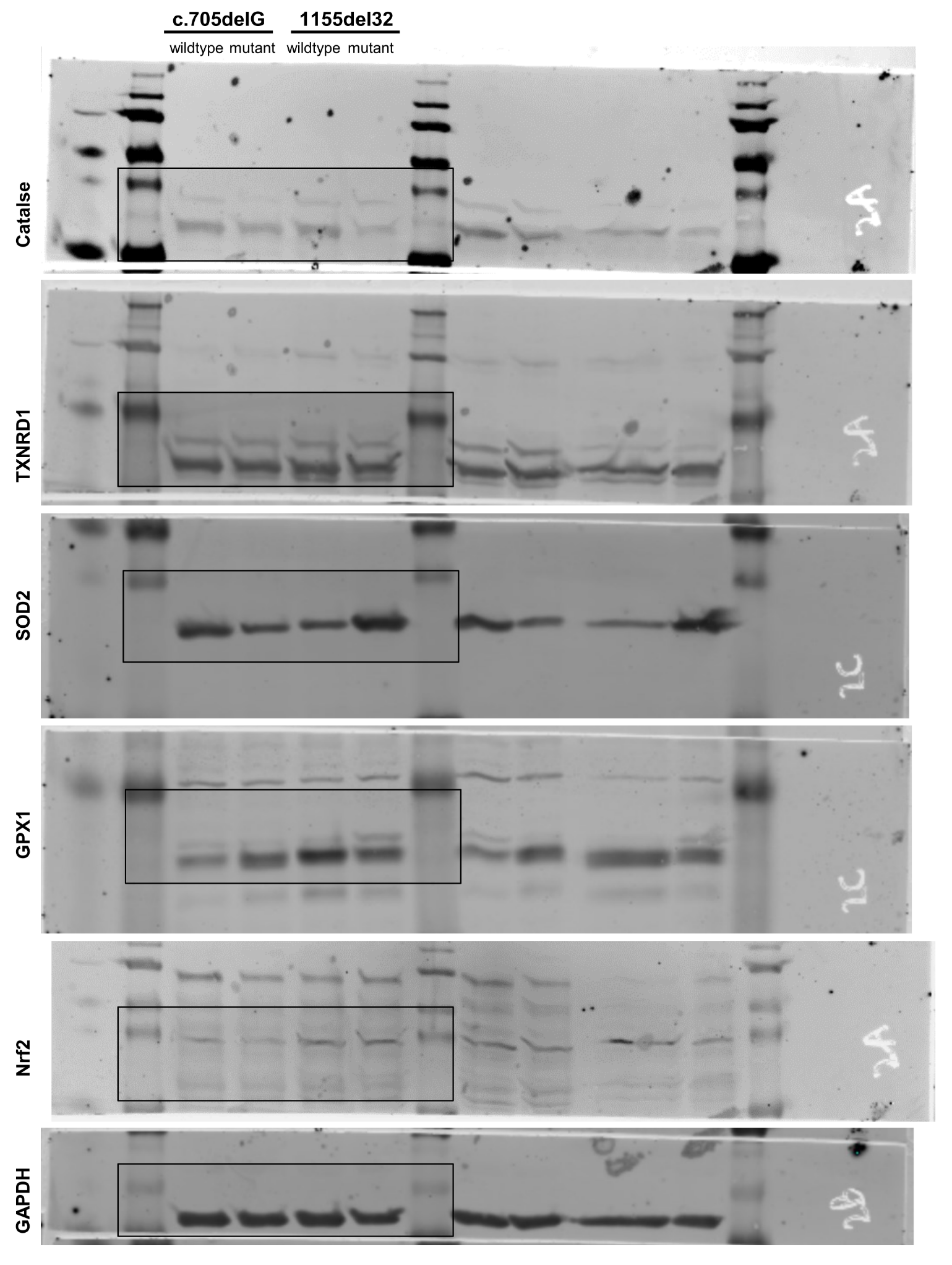


**Supplementary Figure 5. Uncropped images of the immunoblots shown Fig. 4b.**
